# The Involvement of Arginase and Nitric Oxide Synthase in Breast Cancer Development: Arginase and NO Synthase as Therapeutic Targets in Cancer

**DOI:** 10.1155/2018/8696923

**Published:** 2018-04-30

**Authors:** Nikolay Avtandilyan, Hayarpi Javrushyan, Gayane Petrosyan, Armen Trchounian

**Affiliations:** ^1^Laboratory of Biochemistry, Research Institute of Biology, Faculty of Biology, Yerevan State University, Yerevan, Armenia; ^2^Department of Biochemistry, Microbiology and Biotechnology, Faculty of Biology, Yerevan State University, Yerevan, Armenia

## Abstract

It is well established that, during development of malignancies, metabolic changes occur, including alterations of enzyme activities and isoenzyme expression. Arginase and nitric oxide (NO) synthase (NOS) are two of those enzymes considered to be involved in tumorigenesis. The goal of this article was to study the involvement of arginase and NOS in the development of different stages of breast cancer. Our results have shown that human serum arginase activity and NO (resp., and NOS activity) and polyamines quantities increased in parallel with cancer stage progression and decreased after neoadjuvant chemotherapy. For breast cancer, the only isoenzyme of arginase expressed in serum before and after chemotherapy was in a cationic form. The data of Lineweaver-Burk plot with a *Km* value of 2 mM was calculated, which is characteristic for human liver type isoform of arginase. During electrophoresis at pH 8.9, the enzyme exhibited high electrophoretic mobility and was detected near the anode. The presented results demonstrated that arginase in human serum with breast cancer and after chemotherapy is not polymorphic. We suggest that arginase and NOS inhibition has antitumor effects on cancer development, as it can inhibit polyamines and NO levels, a precursor of cancer cell proliferation, metastasis, and tumor angiogenesis.

## 1. Introduction

Breast cancer is one of the most common types of cancer accounting for 19% of all cancer-related mortalities in women. Therefore, it is important to continue improving current diagnostic and treatment tools and to determine new prognostic variables. Research over the last years has convincingly demonstrated an important role for arginase and nitric oxide (NO) synthase (NOS) in tumor immunobiology [1, 2]. Earlier reports focused on the expression of arginase and NOS in murine or human primary cancer tissue as well as malignant cell lines and emphasized its potential role in the promotion of tumor growth via polyamine synthesis or downregulation of NO-mediated tumor cytotoxicity [3, 4]. L-Arginine is used as a substrate by both NOS and arginase to produce NO and urea, respectively. Arginase (EC 3.5.3.1) is a hydrolase, which catalyzes the hydrolysis of L-arginine into L-ornithine and urea [5, 6]. Due to the generation of L-ornithine, arginase is involved in several important downstream metabolic pathways. L-Ornithine can be further metabolized to polyamines (putrescine, spermidine, and spermine) via ornithine decarboxylase (ODC) [7]. The hepatic arginase, arginase I, is a cytosolic enzyme that catalyzes the fifth and final step in the urea cycle, a series of biochemical reactions in mammals during which the body disposes of harmful ammonia. Extrahepatic arginase, arginase II, is a mitochondrial isoform, and its biological function has been the subject of considerable interest. It seems that this isoform provides a supply of ornithine, a crucial metabolite in biosynthesis of glutamic acid, proline, and polyamines.

Currently, there is a rapid growth of polyamines (spermine, spermidine, and putrescine) quantity in blood serum and urine during malignant tumors in different organs [8]. The large quantity of polyamines allows the oncogenes to overcome the immune system and allows the cancer cells to metastasize. Since polyamines are vital for cell proliferation, it is possible that the increased level of ornithine, due to the elevated arginase activity, may be linked to the development of carcinogenesis. Increased polyamine uptake by immune cells results in decreased production of tumoricidal cytokines and the amount of adhesion molecules, and these eventually attenuate the cytotoxic activities of immune cells.

NO is an unorthodox messenger molecule, which has numerous molecular targets. It is the smallest signaling molecule known, which is produced by three isoforms of NOS (EC 1.14.13.39) [2]. Data indicate that NO is the integral part of human physiological response to hypoxia. It has been shown that the concentration of NO biomarkers in blood plasma (nitrite, nitrate) increases in high altitudes at acclimatization. Various oxides of nitrogen are involved in the increase of NO, which provides tolerance for hypoxia [9]. High-altitude residents produce more nitric oxide than low dwellers, which is expressed as a high concentration of NO in the exhaled air, as so by increased level of NO metabolites in blood (nitrites, nitrates, and nitroso compounds). The importance is increasing, taking into account the fact that breast cancer patients with metabolic dysregulation are associated with poor response to current chemotherapy [10].

Whereas Armenia is a high-altitude locality, it was interesting to study the quantity change of total nitrite anions in blood serum during the diseases and after chemotherapy, for revealing the specific regional phenomena. Moreover, according to the World Health Organization, Armenia has a high smoking percent, stress level, and coronary heart disease which also can be responsible for the triggering of NO production (there is no clear data for the increase in nitric oxide concentration). Furthermore, NO either facilitates cancer-promoting characters or acts as an anticancer agent. The dilemma in this regard still remains unanswered. It is important once again to examine and understand that different actions of NO in breast cancer at the molecular level can help in providing NO based diagnostic or prognostic markers and also in devising potential strategies for prevention and treatment.

Increased NO generation in cancer cells may contribute to tumor angiogenesis by upregulating vascular endothelial growth factor (VEGF), and VEGF-induced neovascularization may increase the tumors' metastatic ability. Increased amounts of NO have been observed in the blood of breast cancer patients and higher NOS activity was found in invasive breast tumors when compared with benign or normal breast tissue [2, 11]. NO increases tumor blood flow and promotes angiogenesis, which could explain the positive correlation between NO biosynthesis and grade of malignancy. Although several reports have addressed the protumoral effects of NO, few have demonstrated the contrasting role of NO in mediating tumor regression [12]. Although these tumoricidal roles of NO have been proposed, most experiments have been performed in vitro and such findings have not been reported in cancer patients. It has been suggested that NO concentrations found in tumors are insufficient to produce apoptosis and other tumoricidal effects and are likely to facilitate angiogenesis and tumor dissemination.

The levels of arginase activity and NO and polyamines quantity in malignant tissues have been reported by several researchers to be increased compared with healthy tissues. However, besides the limited number of these studies, none of the authors traced the relation between arginase isoenzymes activity and biological behavior of tumors and NO and polyamines quantity. Therefore, the present study was designed not only to determine arginase activity, nitrite anions, and polyamine concentrations levels in blood serum of breast cancer patients, but also to correlate them with biological behavior of this tumor.

The goal of this article was to study the involvement of arginase and NOS in breast cancer development by the change of polyamines and NO quantities. Arginase activity and polyamines and NO quantity in different stages of breast cancer and after breast cancer stage II patients' chemotherapy in blood serum were investigated. Serum arginase isoenzymes activity and protein quantity by gel and ion-exchange chromatographies were also studied.

## 2. Materials and Methods

### 2.1. Patients

This study was performed with blood serum of patients with breast cancer who were hospitalized at the National Center of Oncology named after V. A. Fanarjyan (Yerevan, Armenia). It included women with primary breast cancer who were hospitalized between 2014 and 2017. None of the patients had received preoperative radiotherapy or chemotherapy (besides patients with breast cancer stage II). None of the patients had other diseases. The Tumor-Nodes-Metastasis (TNM) system was used to describe the cancer stages (American Joint Committee on Cancer) (Table 1) [13]. The surgical intervention and also the chemotherapy (chemo) after surgery on the patients in different stages of disease development were not taken into account. For all women in chemotherapy group, neoadjuvant chemotherapy is used. The drugs used for neoadjuvant chemotherapy include doxorubicin and epirubicin, paclitaxel and docetaxel, 5-fluorouracil (5-FU), cyclophosphamide (Cytoxan), and carboplatin (Paraplatin). Most often, combinations of 2 or 3 of these drugs were used. In our experiments, blood serums were used after total period of treatment. Neoadjuvant chemotherapy was given for a total of 3 to 6 months, depending on the drugs used. All work was conducted in accordance with the Declaration of Helsinki (1964). The study was approved by the Bioethics Committee of Armenia, and informed consent was obtained from all patients.

### 2.2. Chemicals

Chemicals for determination of arginase activity, proteins and NO quantity, electrophoresis, and TLC were obtained from Sigma-Aldrich Co. Ltd. (Taufkirchen, Germany) and Carl Roth GmbH + Co. KG (Germany).

### 2.3. Separation of Serum Arginase

For purification of the enzyme, the procedure described by Porembska et al. for human liver and erythrocytes was used with some modifications [14]. Separation of arginase isoenzymes was performed by the method of Kossman with some modifications [15, 16]. The column (2 × 40 cm) containing Sephadex G-150 was balanced with Na-phosphate buffer (pH 7.2) and 40 fractions each one of 4 ml were collected. 4 ml of low-molecular-weight protein fraction after gel filtration was passed through the CM-cellulose column (CM52, 1 × 18 cm), balanced against 5 mM Tris-HCl buffer, pH 7.2, with elution speed of 24 ml/h, where 32 fractions each one of 4 ml were collected. The peak of arginase activity was adsorbed on the column with a linear KCl gradient (0.05–0.25 M).

### 2.4. Kinetic Properties of Serum Arginase

The double-reciprocal plot was used for analyzing *Km*. Plotting the reciprocals of the Michaelis-Menten data pointed yields a “double-reciprocal” or Lineweaver-Burk plot. To reveal *Km*, the dependence of arginase isoenzymes on different concentrations of the substrate (0.1, 0.25, 0.4, 0.5, 1, 1.5, and 2 mM L-arginine) was determined. Velocities are expressed as *μ*M of urea produced in 1 sec per ml fraction (after gel chromatography, the fractions from 100 to 124 ml).

### 2.5. Polyacrylamide Gel Electrophoresis (PAGE)

PAGE was performed according to the method of Porembska et al. [14]. Electrophoresis was at 16–20°C and 100–120 V for 4 h, at pH 6.7 through the stacking gel (14% w/v polyacrylamide), and at pH 8.9 through the resolving gel (7.7% w/v polyacrylamide). Electrophoresis was performed in Tris-glycine buffer. Gels were stained using 200 ml of the fixative solution containing 0.1% (w/v) Coomassie Brilliant Blue R250 for 1 h with shaking. Gels were subsequently destained in 10% (v/v) acetic acid for approximately 12 h with shaking. Arginase activity was determined in 5 mm gel slices eluted with NaOH-glycine buffer, pH 9.5.

### 2.6. Determination of Arginase Activity

Arginase activity was determined by the colorimetric method of Van Slyke and Archibald with some modifications [14, 16]. 1.5 ml 0.2 M glycine buffer (pH 9.5), 0.5 ml blood serum, 0.2 ml 5 *μ*M MnCl_2_, and 0.4 ml 50 *μ*M L-arginine were added in test tubes. In the supernatant, the final product of the catalysis was determined, which was urea. 1 ml supernatant and 0.25 ml 3% (w/v) diacetyl monoxime (DAMO) were added and boiled in a water bath for 45 min. The intensity was measured with a spectrophotometer at 487 nm. Activity of enzyme was evaluated with the received urea in micromoles in 1 sec (kat).

### 2.7. Dansylation and Thin Layer Chromatography (TLC) Analysis

The method of Seiler was used with some modifications, as follows [16, 17]. Tissues were extracted in 0.2 M cold HClO_4_ at a ratio of about 100 mg/ml. After extraction for 1 h in an ice bath, samples were centrifuged for 20 min at +4°C and 11.500 g (*r*_av_ = 11 cm). 200 *μ*l of HClO_4_ extract was mixed with 400 *μ*l of dansyl chloride (5 mg/ml in acetone), and 200 *μ*l of saturated sodium carbonate was added. Dansyl polyamines were extracted in 0.5 ml benzene and vortexed for 30 sec. Up to 50 *μ*l of dansylated extract was loaded on the preadsorbent zone of silica gel plates, and the chromatogram was developed for about 2 h with chloroform-triethylamine (25 : 2 v/v) solvent system. The dansyl polyamine bands were scraped, eluted in 2 ml ethyl acetate, and quantified in 505 nm. The quantity of polyamines is presented in nM polyamines in 1 ml of serum.

### 2.8. Griess Assay for NO Quantity

Nitrite was measured by the Griess assay, as described [16, 18]. Briefly, 100 *μ*l Griess reagent was added to 100 *μ*l of each of the above supernatants. The plates were read at 550 nm against a standard curve of NaNO_2_. The values were corrected for the NO^2−^ + NO^3−^ content of water, and the recovery of NO^2−^ was calculated.

### 2.9. Protein Quantity

Protein quantity was determined according to Lowry method [19].

### 2.10. Data Processing

Results were expressed as means ± SD and evaluated by Student's *t*-test (single sample) using Statistica software (StatSoft 10.0).

## 3. Results and Discussion

In our research work, arginase activity and polyamines and NO quantities were determined in blood serum of 11 healthy individuals (female, 34–63 years old), patients with breast cancer (54 patients, female, stages I–III, 43–66 years old), and 13 patients after chemotherapy (stage II, 39–63 years old) (Table 1).

A low level of arginase is normally present in plasma of healthy individuals but can become elevated in certain conditions or diseases. As plasma arginase activity is not routinely assayed by clinical chemistry laboratories, the full range of conditions in which it becomes elevated is not yet known [6].

**Figure 1:**
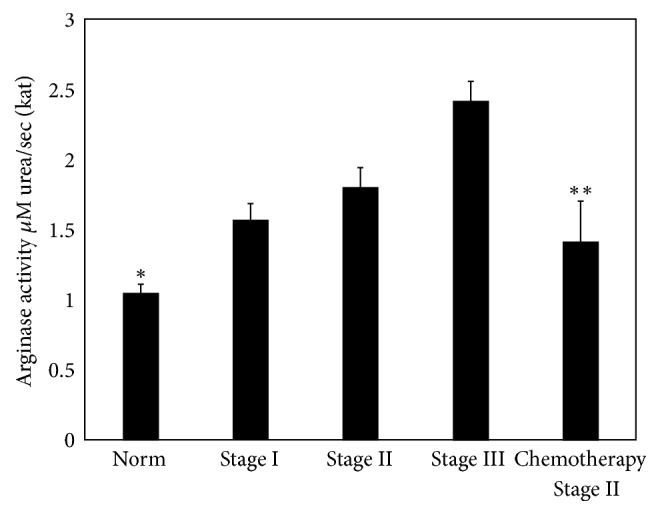
The change of arginase activity in blood serum during different stages of breast cancer and after chemotherapy of patients in stage II, *p* < 0.05. *n* is the number of patients; for *n*, see Table 1; for others, see Materials and Methods; ^*∗*^*p* < 0.001, ^*∗∗*^*p* < 0.05.

Our studies have shown that, in women from the breast cancer group of stage I, activity of serum arginase was increased by 50%, in the group of stage II by 72.1%, and in the group of stage III by 131.7% compared to the healthy group (11 healthy individuals) (Figure 1). This is an interesting finding, since obtained data show statistically significant correlation for all stages in contrast to other published data, where correlations were found only in advanced stages [6, 9, 14, 20]. These results approve that arginase activity increase corresponded to breast cancer progression. An increase of arginase activity was found in 70.6% of patients (true positive) (Table 1).

Special attention was worth giving to the decrease of arginase activity after chemotherapy. In postchemotherapy group (stage II), arginase activity showed 35.6% decrease compared with breast cancer stage I patients (see Figure 1).

Arginase activity after chemotherapy had closer values to stage I; however, it did not reach the control group value. This insinuates that arginase might have some role in promoting tumor growth and development, and its activity can be considered as an important marker to determine disease progression or regression. It is difficult to follow up and control the changes of biochemical options in blood serum in the same patients after half and/or one year after chemotherapy. Therefore, we have scant data processing, which is not reliable and cannot be submitted in this work. Later, with the necessary amount of data, it can serve as a material for a further article.

The calculated values of arginase activity test sensitivity [TP/(TP + FN)], specificity [TN/(TN + FP)], and accuracy [(TP + TN)/(TP + FP + FN + TN)] in serum of women with breast cancer were 83%, 81%, and 82.6%, respectively (see Table 1).

**Figure 2:**
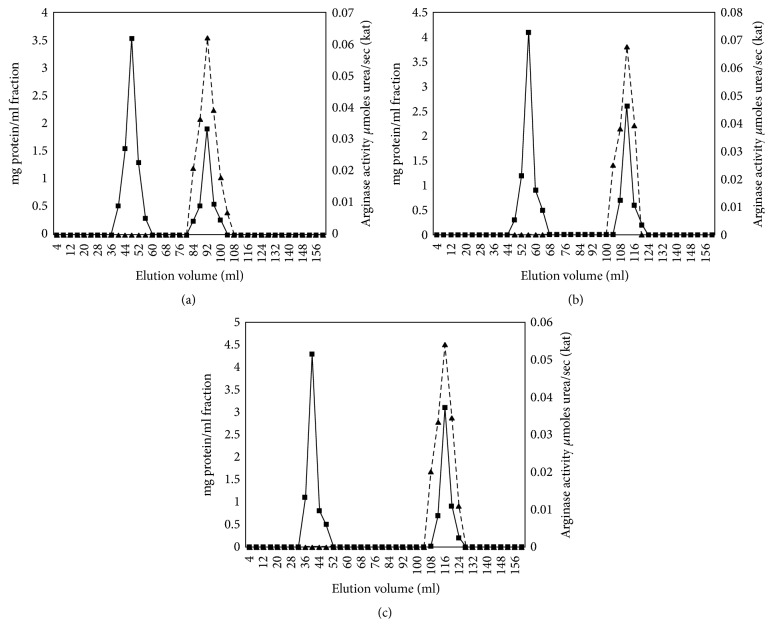
Arginase activity (▲) and protein quantity (■) curves after gel chromatography of serum proteins in healthy individuals, breast cancer patients, and patients after chemotherapy (stage II, *n* = 7, *p* < 0.05). (a) Healthy, (b) breast cancer, and (c) chemotherapy. The enzyme obtained after several steps of purification (see Materials and Methods; 45 mg, 51 mg, and 52 mg protein, resp., for healthy individuals, breast cancer patients, and patients after chemotherapy) was applied to a Sephadex G-150 column (2 × 40 cm).

In blood serum of control group (healthy), gel chromatography (GC) revealed 1 peak for arginase activity (*Ve* = 92 ml) and 2 peaks for protein quantity (*Ve* = 48 and 92 ml) (Figure 2(a)).

In breast cancer group stage II and after chemotherapy, arginase activity was distributed in one (*Ve* = 116 ml) and protein quantity was distributed over two peaks (*Ve* = 52 and 116 ml) (Figures 2(b) and 2(c)).

**Figure 3:**
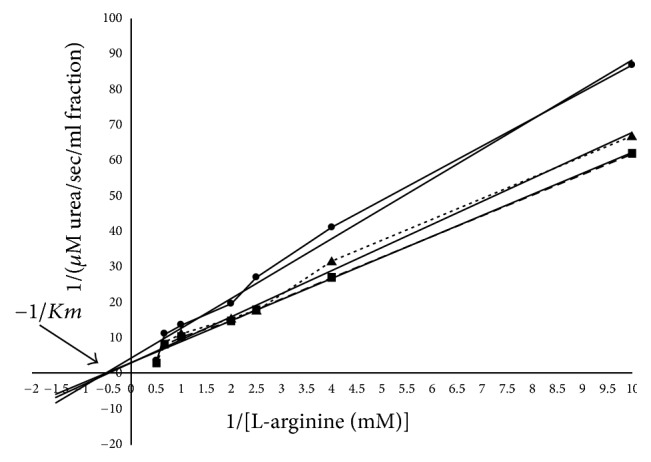
Lineweaver-Burk plot of human serum arginase [serum proteins obtained (9, 11, and 13 mg, resp., for healthy individuals (▲, *Ve* = 92 ml), breast cancer patients (■, *Ve* = 116 ml), and patients after chemotherapy (●, *Ve* = 116 ml)) after gel chromatography was used].

The influence of substrate concentrations, varying from 0.1 to 2 mM, on enzymic activity (fractions after gel chromatography, 92–116 ml, for each group *n* = 7, *p* < 0.05) was studied in 0.2 M glycine buffer (pH 9.5) and 0.2 ml 5 *μ*M MnCl_2_. From this data, a Lineweaver-Burk plot with a *Km* value of 2 mM was calculated, which is characteristic for the liver and erythrocytes type isoform of arginase (arginase I) (Figure 3).

**Figure 4:**
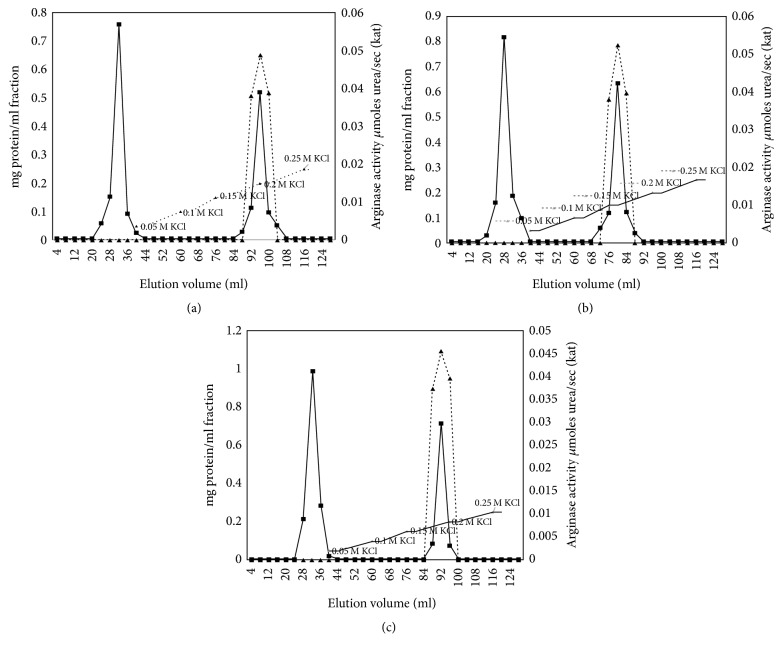
Arginase activity (▲) and protein quantity (■) curves after CM-cellulose chromatography of human serum arginase (stage II, *n* = 7, *p* < 0.05). (a) Healthy, (b) breast cancer, and (c) chemotherapy. The enzyme obtained after gel chromatography (9, 11, and 13 mg protein, resp., for healthy individuals, breast cancer patients, and patients after chemotherapy) to a CM-cellulose column (1 × 18 cm) equilibrated with 5 mM Tris-HCI, pH 7.5, eluted with a KCl concentration gradient. Fractions (4 ml) were collected at a flow rate of 24 ml/h.

**Figure 5:**
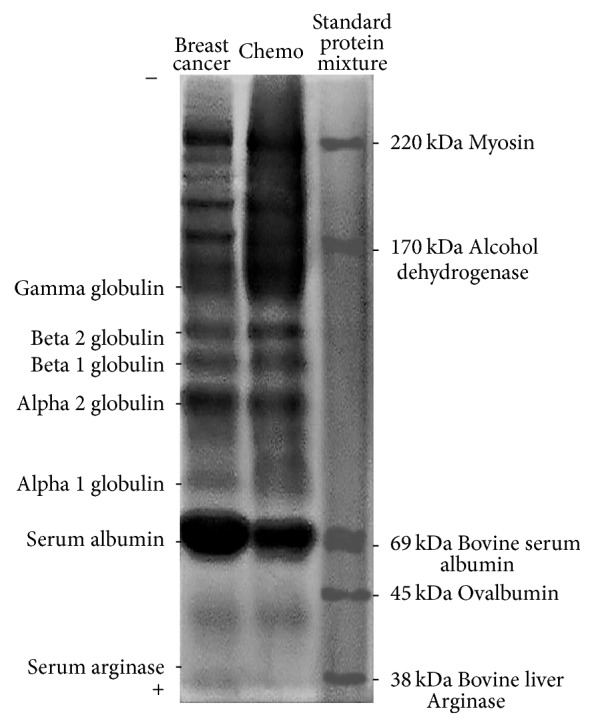
Diagram of electrophoretic patterns of human serum proteins obtained (20 *μ*g) after gel chromatography was used (stage II). Standard protein mixture (each of 10 *μ*g) lane containing molecular weight standards from Sigma: 38 kDa bovine liver arginase, 45 kDa ovalbumin, 69 kDa serum albumin, 170 kDa alcohol dehydrogenase, and 220 kDa myosin.

To confirm this fact, ion-exchange chromatography (IEC) of the second peak fraction (low molecular fraction, *Ve* = 92 and 116 ml) was performed. In ion-exchange chromatography, arginase activity curve is presented with 1 cationic isoenzyme peak (*Ve* = 80 ml); in postchemotherapy group, 1 peak was also visible (*Ve* = 92 ml) (Figures 4(b) and 4(c)). At this point, very few papers are available regarding isoenzyme spectrums in serum. For breast cancer, it was revealed that the only isoenzyme expressed in serum before and after chemotherapy was a cationic form (arginase I) (Figures 3–5).

Our studies have shown the increase of arginase activity in fractions after gel and ion-exchange chromatography compared to the control group and the decrease in gel and ion-exchange chromatography fractions after chemotherapy compared to the cancer group fractions (see Figures 2 and 4). In women with breast cancer, arginase activity (kat) in GC fraction accounted for 0.068 ± 0.007 kat (increased by 9.7% compared with norm, stage II, *n* = 7, *p* < 0.05, Figure 2(b)), and after IEC it is 0.052 ± 0.005 (increased by 5.8% compared with norm, stage II, *n* = 7, *p* < 0.05, Figure 4(b)). The activity in serum from healthy women was lower and it accounted for 0.062 ± 0.006 (*p* < 0.05; after IEC it is 0.049 ± 0.006; Figures 2(a) and 4(a)). The activity in serum from women after chemotherapy was lower than norm and cancer group women and it accounted for 0.054 ± 0.003 (decreased by 20.6% compared with breast cancer group, stage II, *n* = 7, *p* < 0.05, Figure 2(c)), and after IEC it is 0.045 ± 0.007 (decreased by 8.2% compared with breast cancer group, stage II, *n* = 7, *p* < 0.05, Figure 4(c)).

Ion-exchange protein purification is possible because most proteins bear nonzero net electrostatic charges at all pH values except at pH = p*I* (isoelectric point). At pH > p*I* of a given protein, that protein becomes negatively charged (an anion), but at the pH < p*I* of that same protein, it becomes positively charged (a cation). Mammalian liver arginases tend to have basic isoelectric point (p*I*) values of about 8.8–9.4 [21].

Figure 5 illustrates polyacrylamide gel electrophoresis of human serum arginase after gel chromatography (2nd protein quantity peak, *Ve* = 116 ml). During electrophoresis at pH 8.9 (at pH 8.9, most proteins become negatively charged), the enzyme exhibited high electrophoretic mobility and was detected near the anode. Arginase activity for cancer and chemo group was found in the first lane (see Figure 5, serum arginase). This means that p*I* for serum arginase is <8.9. Our results have shown that human serum arginase is a cationic protein, and its mobility at pH 8.9 was similar to the mobility of the arginase form from rat and human liver (see Figures 4 and 5). In human liver, kidney, erythrocytes, and mammary glands, multiple molecular forms of arginase were shown to be present. Our presented results (after ion-exchange chromatography and electrophoresis) demonstrated that arginase in human serum with breast cancer and after chemotherapy is not polymorphic.

Increase of arginase activity allows suggesting that it should have reflection on polyamines quantity. To confirm this assumption in further studies, the quantity of polyamines was determined by thin layer chromatography (TLC). Chromatography results showed that in every stage of cancer along with arginase activity increasing the quantity of polyamines was also growing. The increase of total polyamines quantity in serum compared with standard was 92.6%, 134.3%, 174.2%, and 104.1%, respectively, in stages I, II, and III of breast cancer and after chemotherapy (Table 2).

This coincides with the increase of arginase activity, showing the correlation between them during the disease. The results indicate the acceleration of metabolism as a result of disease, because a large amount of building material is needed, and polyamines are an essential factor for tumor growth.

**Figure 6:**
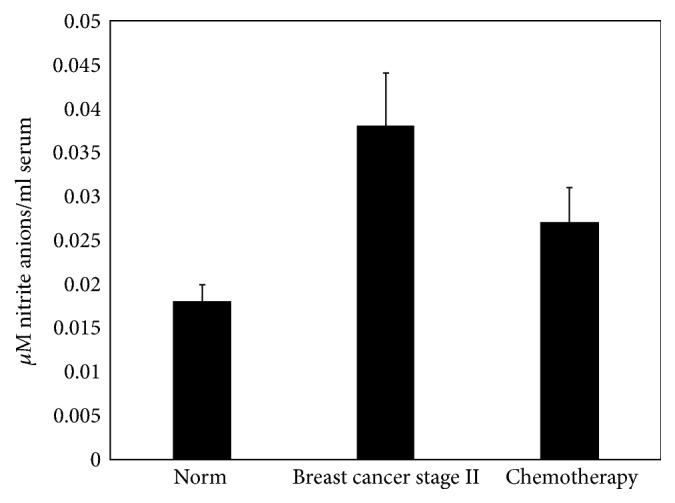
The change of nitrite anions quantity in blood serum of breast cancer patients and after breast cancer patients' chemotherapy of stage II (*n* = 7 for norm, *n* = 11 for cancer of stage II, abd *n* = 7 after chemotherapy, *p* < 0.05).

The results of our study have shown that, in blood serum of patients with breast cancer stage II before chemotherapy compared to the control group, the quantity of nitrite anions was increased by 35.7%, and after chemotherapy it was increased by 14.7% (decreased by 21% compared with cancer group) (Figure 6). Results show that the quantity of nitrite anions in blood serum is rapidly increased during the disease and decreases after treatment. This once again indicates the involvement of NO in the processes of tumor development.

Arginase activity and polyamines and NO quantities are significantly increased during breast malignant cancer, and this enhancement is in correlation with the cancer stages. In the current study, for the first time, it was shown that arginase and NOS activities decrease after chemotherapy, as well as the suggestion was supported, that this enzymes activity was elevated in case of breast malignancies.

## 4. Conclusions and Significance

After chemotherapy, blood serum arginase activity and nitrite anions quantity are decreased, but they still maintain a relatively high level. The decrease of effectivity of chemotherapy and the less sensitivity of patients to it can be related hence to disbalance in L-arginine metabolism, because of the initially high level of NO in blood serum. The last assumption requires further research and a future study will be focused on more thorough examination of the mentioned processes. We can draw a conclusion that arginase and NOS are the important partakers of cancerogenesis and can be considered as an important diagnostic and treatment tools for cancer development. The feature of the arginase and NOS family that makes them attractive as therapeutic targets is that there are small molecules inhibiting these enzymes [1, 10, 20, 21].

The study in our laboratory (electrophoresis, *Km*, and gel and ion-exchange chromatography) show that we have just arginase I in blood serum. These results allow us to narrow the scope of investigation and find noncytotoxic concentration of natural or synthetic specific inhibitor for arginase I. Based on our investigations, we suggest applying direct specific inhibitors for the mentioned enzymes, which will have immediate influence on them, in contrast to other research works, where there is nonspecific chemical's influence on the change of activity of the mentioned enzymes [22, 23]. It should be noted that, in this case, undesirable side effects are possible, which can also activate other metabolic pathways during the diseases.

We suggest that arginase and NOS inhibition may have some antitumor effects on cancer development as it inhibits polyamines and NO levels, a precursor of cancer cell proliferation and tumor angiogenesis. The intermediate of NO synthesis, N^G^-hydroxy-nor-L-arginine (nor-NOHA), is a well-known arginase inhibitor. Noncompetitive inhibition of nor-NOHA and high affinity for the anionic isoform of arginase II create a chance to escape from the possible hyperammonemia due to arginase activity inhibition. N^G^-nitro-L-arginine methyl ester (L-NAME) is one of the most clinically developed NOS inhibitors [10]. Collectively, these observations support the preclinical evaluation of nor-NOHA and L-NAME for the treatment of different types of cancer. Since modern medicine has no effective cure for the malignant cancers and tumors, scientists are interested in finding a potent agent with noncytotoxic properties.

Currently, we study the anticancer influence of different concentrations of N^G^-hydroxy-nor-L-arginine and N^G^-nitro-L-arginine methyl ester (inhibiting arginase and NOS activity correspondingly) in an experimental model of rats with breast cancer induced by 7,12-dimethylbenz(a)anthracene (DMBA). At this stage (studies are in progress), studies show long-term anticancer effect of nor-NOHA and L-NAME. This was mainly manifested by the decrease of tumor sizes and quantity, as well as mortality, and also the biochemical parameters for quantity of polyamines, NO, malondialdehyde (MDA), and histopathological alterations were close to the healthy rats. The obtained results can serve as a base to use our model for determination of productive and noncytotoxic antitumor concentration of anticancer agents. Perspectives of targeting arginase and NOS in cancer management can ground application in clinical medicine. It can also be used to reveal new inhibiting model of enzymes by experimental and theoretical ways.

## Figures and Tables

**Table 1:** Characteristics of patients and arginase activity test in blood serum.

Patients number	Age	Stage	TNM	Characteristic of arginase activity test	Number of patients	%
11	49 ± 15	Healthy	-			

15	57 ± 8	I	T_1_N_0_M_0_, T_1_N_1mi_M_0_, T_0-1_N_1mi_M_0_	True positive (TP)	53^*∗*^	70.6

21	53 ± 10	II	T_0_N_1_M_0_, T_1-2_N_0-1_M_0_, T_2-3_N_0-1_M_0_	False positive (FP)	2	2.8

18	55 ± 11	III	T_0-2_N_2_M_0_, T_3_N_1-2_M_0_, T_4_N_0-2_M_0_, T_any_N_3_M_0_	True negative (TN)	9	12

13	51 ± 12	II Chemo	T_0_N_1_M_0_, T_1-2_N_0-1_M_0_, T_2-3_N_0-1_M_0_	False negative (FN)	11	14.6

^*∗*^3 women in chemo group also involved in cancer group.

**Table 2:** The changes of polyamines quantity (SPM: spermine; SPD: spermidine; PUT: putrescine) in blood serum during different stages of breast cancer (*n* = 10 for each stage, *p* < 0.001) and after chemotherapy in breast cancer patients in stage II (*n* = 7, *p* < 0.05).

Stage of cancer	Polyamine	nM Polyamines/ml serum	
		Breast	Chemotherapy
Norm	PUT	7.2 ± 0.53	-
	SPD	8.6 ± 0.74	-
	SPM	11.3 ± 0.9	-

I	PUT	14.2 ± 1.1	-
	SPD	17.4 ± 1.9	-
	SPM	20.6 ± 1.8	-

II	PUT	17.8 ± 1.7	15.3 ± 1.5
	SPD	21.3 ± 1.2	16.4 ± 1.8
	SPM	24.4 ± 1.7	23.6 ± 2.1

III	PUT	19.9 ± 1.1	-
	SPD	22.6 ± 1.3	-
	SPM	31.8 ± 2.2	-

*R*_*f*_ values for serum polyamines in healthy individuals are *R*_*f*_ SPM = 0.29, *R*_*f*_ SPD = 0.45, and *R*_*f*_ PUT = 0.72; for serum polyamines of the group of women with breast cancer are stage I *R*_*f*_ SPM = 0.3, *R*_*f*_ SPD = 0.42, and *R*_*f*_ PUT = 0.67; stage II *R*_*f*_ SPM = 0.33, *R*_*f*_ SPD = 0.42, and *R*_*f*_ PUT = 0.75; stage III *R*_*f*_ SPM = 0.32, *R*_*f*_ SPD = 0.44, and *R*_*f*_ PUT = 0.65; and after chemotherapy *R*_*f*_ SPM = 0.31, *R*_*f*_ SPD = 0.41, and *R*_*f*_ PUT = 0.68.

## Data Availability

The data supporting the conclusions of this article are included within this article.
